# Head-Mounted Virtual Reality and Mental Health: Critical Review of Current Research

**DOI:** 10.2196/games.9226

**Published:** 2018-07-06

**Authors:** Shaun W Jerdan, Mark Grindle, Hugo C van Woerden, Maged N Kamel Boulos

**Affiliations:** ^1^ Department of Digital Health Centre for Health Science University of the Highlands and Islands Inverness United Kingdom; ^2^ Public Health Directorate NHS Highland Inverness United Kingdom; ^3^ Centre for Health Science University of the Highlands and Islands Inverness United Kingdom; ^4^ The Alexander Graham Bell Centre for Digital Health Moray College UHI University of the Highlands and Islands Elgin United Kingdom

**Keywords:** virtual reality, well-being, behavior change

## Abstract

**Background:**

eHealth interventions are becoming increasingly used in public health, with virtual reality (VR) being one of the most exciting recent developments. VR consists of a three-dimensional, computer-generated environment viewed through a head-mounted display. This medium has provided new possibilities to adapt problematic behaviors that affect mental health. VR is no longer unaffordable for individuals, and with mobile phone technology being able to track movements and project images through mobile head-mounted devices, VR is now a mobile tool that can be used at work, home, or on the move.

**Objective:**

In line with recent advances in technology, in this review, we aimed to critically assess the current state of research surrounding mental health.

**Methods:**

We compiled a table of 82 studies that made use of head-mounted devices in their interventions.

**Results:**

Our review demonstrated that VR is effective in provoking realistic reactions to feared stimuli, particularly for anxiety; moreover, it proved that the immersive nature of VR is an ideal fit for the management of pain. However, the lack of studies surrounding depression and stress highlight the literature gaps that still exist.

**Conclusions:**

Virtual environments that promote positive stimuli combined with health knowledge could prove to be a valuable tool for public health and mental health. The current state of research highlights the importance of the nature and content of VR interventions for improved mental health. While future research should look to incorporate more mobile forms of VR, a more rigorous reporting of VR and computer hardware and software may help us understand the relationship (if any) between increased specifications and the efficacy of treatment.

## Introduction

### Development of Virtual Reality

Virtual reality (VR) is emerging as one of the key new technological tools in a digital revolution sweeping across the health care industry. Immersive VR allows users to interact with a computer-generated world, where the users natural sensory perceptions are replaced with a digital three-dimensional (3D) alternative [1]. To create an immersive VR system, a computer is used to generate an image, a display system is required to project the image, and, finally, a tracker is required to update the image based on users’ movements. Traditionally, VR has been confined to laboratories as expensive and powerful computer(s) are needed to power it. VR, as we know it today, has been around for decades; the vision of VR was first realized by Ivan Sutherland in 1968 with the “Sword of Damocles” head-mounted display (HMD) and later by Morton Heilig with his multisensory Sensorama [2]. Failed attempts at VR systems by Nintendo (Virtual Boy) and Sega (Sega VR) in the 1990s and a further lack of development in the 2000s had many of its critics arguing that the technology was “dead” [3]. However, as noted by Olson et al [4], the video games industry has driven advances in graphics cards that are able to handle increasingly sophisticated 3D constructed environments. Furthermore, simultaneous developments in mobile phones and HMDs have made VR an accessible commodity for consumers. Lately, HMDs have markedly improved: an increased field of view (FoV), higher-resolution images, lightweight comfortable design, and an appealing price have added to its attraction [5]. Steed and Julier [6] described how they designed an immersive VR system around an Apple mobile phone (iPhone 4s), which had the computing power to act as a controller for a VR system. The implementation of gyroscope technology in mobile phones, which tracks user movements, has allowed HMDs to house mobile phones that act as the VR system itself. An example of the capabilities of gyroscope is the highly successful app “Pokemon Go” that tracks users’ movements as part of an augmented reality experience [7]. Collectively, these developments have brought VR back into the public domain. Furthermore, low costs, innovative apps, and an increasing accessibility have captured the imaginations of researchers who have proposed its use in the treatment and assessment of a wide range of health care issues.

### Building the Case for a Review

Evidently, VR is a rapidly emerging field of research. Since 2016, new HMDs from Oculus, HTC, HP, Acer, Dell, and Sony and the arrival of a range of cheaper mobile phone alternatives have acted as a catalyst for a new wave of VR research. Despite recent investment in Oculus by Facebook, indicating VR is here to stay, its popularity among consumers is unlikely to affect the quantity of research around it. VR research has been continuously expanding in a time where it has not been at the forefront of digital consumerism. Oculus’ chief scientist Michael Abrash has suggested that in the next 5 years, we will see a new generation of VR products, which will operate with 4k screen resolution and with eye tracking that may allow for foveated rendering [8]. This prediction would appear to be coming partly true as companies race to produce HMDs with increasingly crisper resolution over their competition [9]. This suggests a potential new wave of VR products, thus, bringing down the prices of even the more sophisticated forms of VR today and making it an even more appealing tool for the health industry. With this in mind, now is the time to review the recent VR research, taking a view of what technology is being used and how it is being used.

### Aims of This Literature Review

In this review, we aim to critically assess the current state of head-mounted VR research in relation to mental health. By doing so, we look to determine which conditions are more susceptible to VR interventions, which conditions need more attention, and in what form VR interventions are most effective. Our secondary aim is to understand more about VR used over the past 5 years and compare it to the new generation of VR in terms of accessibility and specifications. As there are indications that VR HMDs can be used at home as a self-help resource to provide a valuable tool for public health, in this review, we will assess head-mounted VR health research to date to determine whether this has been tested.

### Key Concepts

HMD specifications are categorized into FoV, image resolution, and refresh rate (Hz). FoV refers to the view or surroundings a human can see without any eye movement. The human eye has a rotating FoV of up to 270° [10]; newer HMDs are attempting to create a FoV closer to that of the human eye. Currently, we can expect the Oculus Rift and HTC Vive to give an FoV of 110°, while some prototypes such as the Pimax 8k claim to offer an FoV of 200° [11]. Image or screen resolution refers to how clean and crisp the picture quality is; this is determined by the number of pixels in an image area and is reported by the number of pixels arranged horizontally and vertically [12]. For example, a screen resolution of 1280 ⨯ 720, which we refer to as 720p, is classified as high-definition (HD) ready. High-end HMDs today give a resolution between full HD (1080p) and QHD (1440p); again, both the Oculus Rift and HTC Vive offer a screen resolution of 2160 ⨯ 1200, which equates to 2,592,000 pixels per image. This method of reporting resolution has been key to selling televisions, which we see advertized as “full HD 1080p” or “4k.” The investment that companies put into screen resolution can be seen by HTC’s upgrade of the Vive to the Vive Pro, the two HMDs are essentially very similar with exception of the Vive Pro’s increased 2880 ⨯ 1600 resolution. The refresh rate reported as hertz is the number of times a screen can change image. We refer to this refresh rate as frames per second (FPS); an increased FPS will give a more fluid motion of images. FPS is particularly important as we want movements to be realistic; an environment should act according to the user, which means the reduction of any lag between the users’ input and the output of images. Furthermore, VR setups that operate below 90 FPS are more likely to induce nausea and disorientation [13].

We used a useful definition of mental health from a mental health foundation that defined it as:

A state of well-being in which the individual realises his or her own abilities, can cope with normal stresses of life, can work productively and fruitfully, and is able to make a contribution to his or her community.

14

The World Health Organization acknowledges that positive mental well-being is rooted within mental health; this state of well-being allows an individual to lead a fulfilling and productive life [15]. In this review, we aim to look at conditions that offer a scope to deliver psychological change that can make a meaningful difference to one’s mental health. We excluded severe mental illnesses that require a more complex approach to treatment.

### Virtual Reality and Mental Health

Health care and VR first met in the 1990s as a simulation tool for colonoscopy and upper gastrointestinal tract endoscopy simulation within medical education [16]. VR would have remained as a simulation tool for physicians and surgeons, but its interactive nature suggested it as an applicable tool for psychological change. For example, in the therapy of phobias, adaptability of virtual worlds means that contextually relevant virtual worlds can be created that are used to enable systematic exposure to feared stimuli [17]. The ability to precisely control stimuli has allowed VR ecological validity in its assessment of behaviors, emotions, and cognitions [18]. As a result, established effective psychotherapeutic approaches, such as cognitive behavioral therapy (CBT), are recreated within VR alongside exposure techniques [19]. This exposure is particularly effective as the goal of VR is to produce an “illusion of reality” [5]; however, for the patients, despite knowing that the computer environment is not real—a computerized illusion—their brains perceive the images and sound as real stimuli [1]. The broad reach of VR has enabled its use for treating schizophrenia, posttraumatic stress disorder (PTSD), social and generalized anxiety disorders, specific phobias, eating disorders, substance abuse, attention-deficit/hyperactivity disorder, depression, pain management, and psychological stress, as well as its use as part of a wide range of poststroke rehabilitation.

## Methods

### Design

Narrative syntheses were conducted on VR studies that were pertinent to areas of mental health [20]. The literature was critically assessed within the parameters of our review aims. Control and noncontrol studies were included, and studies varied from theory and assessment to treatment. Studies were featured if they appeared in peer-reviewed journals.

### Inclusion and Exclusion

We set an inclusion timeframe from January 2012 to July 2017. A 5-year period was seen as sufficient enough to reflect the current state of the technology; this would allow us to assess studies that used both the new generation of HMDs and a range of older HMDs that have been in academia for the past decade. In line with this, studies were only included if they used an HMD. This meant excluding studies that used Cave Automatic Virtual Environment, the Computer-Assisted Rehabilitation Environment, and other projector systems without an HMD. As we focused on the systems used, if authors failed to disclose the type of VR used, or stated they used an HMD but provided no further information regarding its model or specifications, the study was excluded. Studies involving the use of two-dimensional (2D) virtual environments such as those seen in Second Life were excluded. Furthermore, augmented reality studies were not included as although augmented images are computer generated, the environment itself is not. Future VR may well feature an augmented experience within it [21]; however, at this time, the two technologies are separate, and this is reflected in this study’s direction.

Mental health conditions were categorized as behavioral conditions that showed the potential to be modified upon intervention. In accordance with the International Statistical Classification of Diseases and Related Health Problems [22], we were primarily concerned with the areas of “Neurotic, stress-related, and somatoform disorders,” “Behavioral syndromes associated with physiological disturbances and physical factors,” “Mood disorders,” and substance abuse. We included VR studies that featured any form of anxiety (generalized anxiety disorders, social anxiety disorders or specific phobias, and PTSD), depression, eating disorder (anorexia and binge eating), sleep disorders, and substance addiction or abuse. Pain management was also included as it has profound psychological and emotional consequences that can lead to depression and anxiety [23]. Severe mental disorders were excluded; a number of studies on psychosis were identified; however, due to its neurological origins as a state of brain development rather than a disease, psychosis was excluded from the study [24]. Autism was also excluded due it its neurodevelopmental origin. Finally, we excluded any rehabilitation studies, typically on stroke [25].

### Search Strategy

The search strategy implemented in this review was conducted in 5 stages.

1. Key reviews in the area were identified; these contained broad mental health VR reviews to more condition-specific reviews.
2. The results and reference lists of these reviews were scanned to make an initial list of suitable studies.
3. Our own searchers were then carried out to identify any missed and more recent studies. The terms searched were: [“Virtual”] AND [“mental health” OR “well-being”]. This was followed with more condition-specific searches: [“Virtual”] AND [“Anxiety” OR “Social Anxiety” OR “Phobia” OR “Agoraphobia” OR “Arachnophobia” OR “Fear” OR “PTSD” OR “Depression” OR “Depress” OR “Stress” OR “Abuse” OR “Addiction” OR “pain” OR “Substance” OR “Eating” OR “Disorder” OR “Sleep” OR “Body Image” OR “Body” OR “sexual” OR “Dysfunction”]. Searches were conducted within “MEDLINE,” “Journal of Medical Internet Research,” “PsychINFO,” “Google Scholar,” and “Science Direct.”
4. Screening was carried out upon the completion of a comprehensive list of studies. At this stage, studies were excluded based on publication date, condition type, and lack of immersive VR.
5. The full texts of remaining articles were then assessed to find further reason for exclusion; at this stage, studies were typically excluded for not using an HMD or failing to disclose the type of VR used.

A full list of identified studies can be found in Multimedia Appendix 2.

## Results

### The Current State of Research

#### Head-Mounted Displays

The following findings have been compiled from 81 studies that used HMDs in interventions related to mental health; 18 HMDs appeared across six different areas of mental health. The eMagin z800 (n=34) was the most commonly used form of HMD appearing 34 times (Table 1). At the time of the review, the only quotable price for this product was US $1795, with no stock available in the UK. The z800 operates with a screen resolution of 800 ⨯ 600, a 40° diagonal FoV, and the standard refresh rate of 60 FPS. The second most commonly used HMD was the nVisor sx60 (n=8), which despite a higher screen resolution compared with the z800, still features a relatively low FoV compared with the consumer products used today. Two successful embodiment studies made use of HMDs with higher specifications: an nVisor sx111 with a resolution of 1280 ⨯ 1024 and a 111° diagonal FoV was used to provide compassion to crying baby avatars [26]. Keizer et al [27] used a second-edition Oculus Rift Developers Kit (960 ⨯ 1080 screen resolution and 100° nominal FoV) to reduce the level of body size misestimation among anorexic patients. Interestingly, the Oculus Rift was used in 5 of 22 pain management studies but in none of the anxiety studies, which may reveal a preference for certain specifications for certain medical conditions. Mobile phone VR was used once by Taskian et al who made use of Samsung’s Gear HMD [28].

#### Anxiety

VR has been used as a form of exposure treatment (VRET). Its uses include the following conditions: social anxiety disorder [29-31]; PTSD for military veterans [32] and for World Trade Center attack [33] and assault victims [34]; a range of specific phobias, focusing on fear of flying [35] and fear of spiders [36-39]. Various forms of VRET are featured throughout the anxiety literature. Some studies have compared the efficacy of VRET versus *in vivo* exposure [30], while others have incorporated VRET with CBT and compared it with traditional CBT [29]. The contained exposure that VR provides has led to controlled studies where the effects of cycloserine and alprazolam have been tested for the treatment of PTSD [32,33,40]. Similarly, VR environments have been used for Theta burst stimulation [41] for those with spider phobia. The literature review revealed that VR exposure showed positive results on levels of anxiety and, generally, was at least as effective as *in vivo* exposure, although in some cases, the latter was slightly more effective [30]. A similar trend was noted for VR and CBT studies, with Bouchard et al [29] finding an increased level of effectiveness for VRCBT over traditional CBT. In addition, Malbos et al [42] found that when treating anxiety for agoraphobia, VRET was just as effective in reducing anxiety as VRET with CBT combined. VR has been proven to be a markedly effective tool to induce fear to stimuli [19], and it is also able to predict future levels of PTSD severity [34] and diagnose patients with the condition [43].

#### Pain Management

VR has been used as a distraction tool for pain management. Different forms of VR distractions have been used for burn wound pain [44-47], phantom limb pain [48,49], cold pressor pain [50,51], dental pain [52,53], neck pain [54,55], back pain [56], and cystoscopy [57] as well as for the assessment of analgesia [58-60] and kinesiophobia [56,61]. Distractions ranged from coastal walks [52] and target-aiming tasks [61] to a Sonic the Hedgehog Nintendo video game [51]. Other treatments were dictated by the pain they were treating; visual limb distractions were used for phantom limb pain [48,49]. Bahat et al used VR to manage impairments in cervical kinematics, which is commonplace for those with neck pain [54]. Two studies reported VR distraction to be more effective at reducing perceived pain than controls, which included passive 2D distractions [38,44]. In contrast, Sil et al reported that an interactive video game without VR was equally as effective at reducing pain as the same game with VR [51]. Piskorz et al focused on the varying levels of complexity within VR distraction tasks and concluded that more complex tasks were more effective at reducing the levels of pain [50]. In total, we identified 22 studies; VR delivered through HMDs was frequently seen as the most effective method to distract from pain and, at worst, was equally as effective as controls.

#### Stress

Stress was the primary target and depression the secondary in Shah et al’s VR mood induction procedure study [62]. That study did not have a control group, but the VR-based stress management program did show a decrease in the levels of depression and stress. This result was achieved by face-to-face psychoeducation that centered on relaxation practice and on how to manage stress; relaxation techniques were then practiced in VR. The Trier Social Stress Test (TSST), a paradigm used for inducing psychosocial stress, was successfully implemented in VR [63]. Designed to induce rather than reduce stress, VR-TSST showed a marked increase in peripheral and subjective physiological reactions compared with *in vivo* TSST. Finally, VR-based mindfulness apps may also prove to have a positive effect on stress [64].

#### Depression

We identified only one intervention that had depression as the primary target [36]. Patients delivered a compassionate message to a crying baby avatar and then received the same message while embodied as the baby. After three repetitions of the scenario, patients demonstrated a marked reduction in depression severity and self-criticism, with a substantial increase in self-compassion.

**Table 1:** Head-mounted display (HMD) specifications.

Type			Resolution	Hz	Field of View	Number of Studies
eMagin z800			800 × 600	60 Hz	40°	34
nVisor SX60			1280 × 1024	60 Hz	60°	8
nVisor SX111			1280 × 1024	60 Hz	111°	4
nVisor ST50			1280 × 1024	60 Hz	50°	1
Sony HMZ-T1			1280 × 720	60 Hz	51.6°	1
5DT HMD			800 × 600	N/A^a^	40°	3
VisuaStim			1280 × 1024	85 Hz	40°	1
Kaiser XL 50			1024 × 768	60 Hz	50°	1
VR1280			1280 × 1024	60 Hz	60°	1
Virtual Realities VR HMD pro 3D-42			800 × 600	N/A	42°	1
Pro			640 × 480	60 Hz	71.5°	2
Vuzix iWear VR920			640 × 480	N/A	N/A	5
Vuzix VR1200			N/A	N/A	N/A	2
VFX3D			640 × 480	N/A	35°	1
Sensis Zsight			1280 × 1024	60 Hz	60°	2
V6 by Virtual Research Systems			640 × 480	60 Hz	60°	1
V8 by Virtual Research Systems			640 × 480	60 Hz	60	1
Oculus Rift DK1			640 × 800	60 Hz	110°	1
Oculus Rift DK2			960 × 1080	75 Hz	100°	6
ITV goggles ITG Wideview Xl edition			N/A	N/A	N/A	1
Samsung Gear VR			2560 × 1440^b^	60 Hz	96°	1
i-glasses 920HR			N/A	N/A	35°	1
Kaiser Optics SR80a			N/A	N/A	N/A	1
**Capable HMDs commonly sold on the market today**						
		HTC Vive	2160 × 1200 (combined)	90 Hz	110°	N/A
		HTC Vive Pro	2880 × 1600 (combined)	90 Hz	110°	N/A
		Oculus Go	1280 × 1440 (per eye)	72 Hz	N/A	N/A
		PlayStation VR	1920 × 1080	90 Hz (120 Hz in cinema mode)	100° (approximately)	N/A
	Samsung Odyssey		1440 × 1600 per screen	90-60 Hz	110°	N/A

^a^N/A: not applicable.

^b^Super AMOLED (active-matrix organic light-emitting diode) and dependent on mobile phone used.

#### Eating Disorders

Studies on eating disorders included those on body image disturbance (BID) [65,66], anorexia, and bulimia nervosa [36,67]. Gutiérrez-Maldonado et al [67] compared this form of VR with a more immersive VR that used the Oculus Rift Developers Kit 1. The study found immersive VR to be slightly more effective at reducing food cravings compared with nonimmersive VR. Mountford et al [66] concluded that dieters reported higher social evaluative concerns compared with nondieters. In addition, Purvis et al [65] found that women reported higher levels of body satisfaction in a VR environment than in control conditions. In an innovative study, Keizer et al [27] created a full-body illusion treatment and concluded that disturbed experiences of the body in patients with anorexia nervosa could be altered with VR.

#### Addiction and Substance Abuse

HMDs have been used to deliver exposure therapy to help treat tobacco addiction. Four studies investigated tobacco addiction [68-71]; one focused on relapse prevention of tobacco consumption [72] and one on nicotine dependence [73]. The remaining studies focused on gambling addiction [74,75] and adolescent risk reduction [76]. Virtual reality cue exposure therapy (VR-CET) was used to various degrees of success in the majority of studies. Pericot-Valverde et al [68] found VR-CET to be as effective as traditional CET for smoking cessation and, in a later study [69], stated that VR-CET might be more beneficial toward certain individual variables, particularly age. Forms of CET were used in 6 out of 8 studies, and VR-based cue reactivity assessment approach was used [73] to demonstrate the feasibility of cue exposure within VR to treat nicotine dependence. Lister et al [74] did not test the efficacy of VR to treat gambling addiction but, nevertheless, constructed a VR environment to demonstrate how goal setting within gambling can lead to a chasing behavior that can amount to large financial losses for gamblers. Another study compared the efficacy of CBT against VRET for preventing relapses in nicotine dependence [58].

## Discussion

This review confirms that HMDs have been used to treat mental health in different ways. VRET was one type of intervention that was consistently used across different conditions. VRET interventions with and without CBT content have been implemented for therapy of anxiety, PTSD, stress, eating disorders, and substance addiction. VR excels in its advantage of being able to draw on both audio and interactive visual stimuli, making the fearful stimuli appear as real as possible. In addition, CBT delivered in VR has shown consistent positive results; the accurate adaption of relevant stimuli allows CBT to pinpoint troublesome behaviors. The merging of VR and mobile phones is a timely collaboration, and stress management apps for mobile phones have been described as “incremental acquisitions” to cope with daily-life stresses [77]. The release of cost-effective HMDs, such as Google Cardboard, along with mobile phone–compatible Samsung Gear VR is paving the way for an accessible form of health promotion that encompasses the mobile nature of mobile phones and the interactive exposure of VR. However, this review revealed only one occasion when a mobile HMD was used [38]. A rise in cryptocurrencies has implied that the cost of personal computer (PC) graphics cards that power VR graphics is increasing rather than declining as once expected [78]; moreover, added with the announcement of Oculus Go [79], it highlights the need to demonstrate the clinical capabilities of mobile VR that does not rely on high-end PCs. Future research should focus on testing a VR experience that can be used at home; the cheaper alternatives discussed would be an ideal starting location.

Lindstrom [80] highlighted the work of Aaron Antonovsky’s “Salutogenesis” in which he points to a method of “generating” health and emphasized an important difference between public health and biomedical models: health promotion focuses on the resources toward health over the cautionary tales of risk and disease. Early results in the treatment of stress suggest that VR is an ideal platform to combine exposure to relaxation and provide psychoeducation [62]. There is a need to investigate how the core features of VR can create exposure to positive stimuli that help promote health. VR research into pain management has highlighted how positive VR experiences can provide a pain-relieving distraction. If exposure to pleasant stimuli in relaxing environments is as successful as evoking fear during deliberately troubling environments, VR could prove to be an important novel platform for providing people the resources toward health. While nonimmersive virtual environments were excluded from this review, it is worth noting how health promotion became a prominent feature of the computer program Second Life [81], as users were able to interact with bulletin boards, multimedia productions, power points, health videos, and links to health-related Web pages. These acted similar to psychoeducation in the sense that both information and education were offered [82]. Offering a more immersive experience than Second Life, there is reason to believe that similar health promotion tactics could succeed in a VR environment. Further research is needed to determine whether exposure to positive stimuli is as effective for mental health as exposure to negative stimuli is for a psychological change.

The success of VR in the treatment of anorexia- and depression-focused embodiment studies [27,36] highlights its effectiveness in treating conditions that can be “visual” for the sufferers. While there is no evidence to suggest that newer VR systems—with higher screen resolution and FoV—are more clinically successful than older VR systems, HMDs with higher specs may be better equipped to execute successful condition-focused interventions. For example, the Oculus Rift with its 110° FoV was not used in any of the anxiety studies but appeared in 5 of the 22 pain management studies. This could be attributed to the preferences of the authors; however, it could be argued that the Rift with its larger FoV is more suitable for distraction interventions, whereas this increased FoV is not as crucial when trying to evoke a fearful reaction in someone with anxiety. It is also hard to determine whether VR HMDs are being used to their full potential. The reporting of materials in the reviewed literature was frequently limited, and we even excluded studies for failing to specify the type of VR used; however, the specifications of PCs being used to power VR were even less frequently reported. All HMDs featured in Table 1 allow images to be displayed at least 60 FPS, with newer models allowing for 90 FPS. Many computer systems are unable to reach this potential as the experience of VR being powered by a 2GB Nvidia GeForce GTX 950 graphics card will be different to a newer 11Gb GeForce GTX 1080Ti. This matters because we know that frame rate judder caused by lower FPS can be a catalyst for motion sickness [83]. Thus, we recommend the use of FPS tools, as suggested [84], to provide more rigor to material and method reporting. In the interest of making the research replicable, we also recommend the development of a framework for reporting technical specifications of VR.

The state of research suggests that VR cannot be a clinical tool itself and, instead, its success relies on the content it provides a platform for. Complex VR systems backed by PCs with high graphical and processing power to build detailed and adaptable environments allow the content of the intervention to be complemented further. The most common HMD was the eMagin z800, which in the UK is not an accessible product. In public health context, it is imperative that commonly sold HMDs are used with VR apps that can be used for self-help and to promote health. This review points to VR as a useful method of modifying the behavior in an effort to enhance mental health; the challenge now is to apply this to accessible products, which the public can use at home, work, and on the move.

The results of this review suggest the potential efficacy of VR to provide a platform for improved mental health. VR has demonstrated some compatibility with proven psychological interventions, but combined, they illustrate a potential for a real positive behavior change for a range of mental health conditions. The current state of research does not illustrate VR’s ability to improve mental health on its own; instead, it highlights the importance of the condition-oriented content within VR interventions.

However, the specifications of HMDs and the computers that power them are still important when trying to improve mental health using VR. Although increases in FoV have brought us closer to FoV of the human eye, an improved FPS may decrease the chances of motion sickness for some users. Currently, VR’s strengths are being used for exposure therapy, as successful interventions in the treatment of anxiety, phobias, and PTSD have been demonstrated. In addition, VR-induced distraction has proven to be a remarkable development in pain management. The lack of studies surrounding stress and depression, despite positive initial outcomes, highlights VR’s infancy in some areas of mental health. To help understand more about the relationship between VR systems and its efficacy as a mental health tool, we recommend a thorough reporting of HMD and computer specifications. Finally, there is a need to design interventions that make the most of VR’s increasing mobility, as self-help VR tools could prove to be a valuable asset for mental health services. Thus, researchers must make the most of a rapidly developing medium that is seeing advances in equipment; this will act as a catalyst to develop increasingly detailed and novel interventions that push the boundaries of virtual presence. By achieving this, VR has the potential to radically change the way we modify problematic behaviors that affect our mental health.

## Appendix

Multimedia Appendix 1 Literature table.

Multimedia Appendix 2 Head-mounted display (HMD) virtual reality (VR) use as reported in literature 2012-2017 (n=81).

## Abbreviations

CBT: cognitive behavioral therapy
FoV: field of view
FPS: frames per second
HD: high definition
HMD: head-mounted display
PTSD: posttraumatic stress disorder
TSST: Trier Social Stress Test
VR: virtual reality
VRET: virtual reality exposure treatment
