# Supplementary material for: Head-Mounted Virtual Reality and Mental Health: Critical Review of Current Research
Source: JMIR Serious Games. 2018 Jul 6;6(3):e14. doi: 10.2196/games.9226 (PMC6054705; doi:10.2196/games.9226)
Supplement: Multimedia Appendix 1 [file games_v6i3e14_app1.pdf]

TABLE 1 - HEAD MOUNTED VR LITERATURE 2012-2017 (n=82)

Anxiety Disorders (n=42)

AUTHOR(S)	YEAR	COUNTRY	CONDTION	SAMPLE	INTERVENTIONS	VR USED (HMD alternatives and relevant technologies)
Aymerich-Franch et al	2014	Spain	Anxiety	252	Representative avatars vs non representative avatars for public speaking	nVisor SX111
Bouchard et al	2016	Canada	Anxiety - SAD	59	VR CBT, CBT	eMagin z800
Depperman et al	2016	Germany	Fear of Spiders - Arachnophobia	83	Theta burst stimulation (iTBS) with VR	eMagin Z800
Dibbets et al	2015	The Netherlands	PTSD	39	VRET, Tradtional Trauma Film Paradigm (TFP)	nVisor ST-50
Diemer et al	2013	Germany	Fear of spiders	58	single dose of quetiapine XR, placebo prior to a VR spider challenge	eMagin Z800
Diemer et al	2016	Germany	Fear of flying	80	VRET	eMagin Z800
Difede et al	2014	USA	PTSD (world Trade centre attack)	25	VRET with D-cycloserine, VRET with Placebo	Kaiser XL-50 VR
Freeman et al	2014	UK	PTSD (Physical assault victims)	106	VR assessment of PTSD and paranoia levels	Virtual Research VR1280
Freeman et al	2013	UK	PTSD (Physical assault victims)	106	6 month follow up of PTSD symptoms	Virtual Research VR1280
Glotzbach-	2013	Germany	Anxiety	49	VR office with	eMagin Z800

Schoon et al					electrical stimuli (CXT+), VR office without electrical stimuli (CXT-)	
Hartanto et al	2014	The Netherlands	Anxiety – SAD	16	VR scenarios (neutral, blind date, job interview)	eMagin Z800
Kampmann et al	2016	The Netherlands	Anxiety - SAD	60	VRET, iVET, Waiting List	nVisor SX60
Levy et al	2016	France	Fear of heights	6	VRET	Sony HMZ-T1
Levy et al	2016	France	Phobia falling	16	VR games	V8 HMD, EyeToy interface for PlayStation 2, Video projector
Malbos et al	2013	Australia	agoraphobia	19	VRET, VRET with CBT	Virtual Realities HMD Pro 3D-42
Meyerbroeker	2012	The Netherlands	Fear of flying	67	VRET with Yohimbine Hydrocholoride (YHO), VRET with Placebo	Cybermind Visette Pro
Morina et al	2014	The Netherlands	Anxiety	43	VR with varying levels of presence	Nvisor SX 60
Morina et al	2015	The Netherlands	Anxiety	43	VRET	nVisor SX 60
Mosig et al	2014	Germany	Arachnophobia	43	VR differential fear conditioning paradigm, paired with either high frequency tone CS+ and low frequency tone CS-	eMagin z800
Muhlberger et al	2012	Germany	Fear of heights	56	Depth and belief cues manipulated in VR	eMagin z800
Norrholm	2016	USA	PTSD	50	VR PTSD	eMagin Z800,

					treatment with Administered D-cycloserine (DCS), alprazolam (ALP), or placebo (PBO)	
Notzon et al	2015	Germany	Arachnophobia	83	intermittent theta burst stimulation (iTBS) applied prior to VR experience.	eMagin Z800,
Pallavicini et al	2013	Italy	Anxiety	39	Text (TX), Audio (AU), Video (VD), VR	Vuzix VR Bundle, Xbox controller.
Peperkorn et al	2016	Germany	Fear of spiders	64	VR fear exposure	eMagin Z800,
Peperkorn et al	2014	Germany	Fear of spiders	96	VR perceptual cues	eMagin Z800,
Powers et al	2013	USA	Anxiety - SAD	26	VRET, In vivo	eMagin z800
Price et al	2015	USA	PTSD	116	VRET with *D-Cycloserine 50mg or alprazolam 0.25mg, compared to placebo*	eMagin Z800
Reger et al	2016	USA	PTSD	162	Prolonged exposure vs VRE	eMagin z800
Rinck et al	2015	The Netherlands	Fear of spiders	63	Immersive Virtual Environment (IVE)	Nvisor SX60,
Rothbaum et al	2014	USA	War veterans with PTSD	156	VRET + D-cycloserine, VRET + alprazolam, VRET + placebo	eMagin Z800
Rus-Calafell	2013	Spain	Fear of Flying	15	Imaginal exposure, VRET	HMD 5DT model

Safir et al	2012	Israel	Public Speaking Anxiety	88	VRET + CBT CBT Waiting list	VFX3D from Interactive Imaging Systems
Shiban et al	2016	Germany	Claustrophobia	96	VR fear reactions, specific cues (C), fear related information (I) and both.	eMagin Z800
Shiban et al	2016	Germany	Spider phobia	61	VRET	eMagin z800
Shiban et al	2015	Germany	Spider phobia	32	VRET, in vivo	eMagin Z800
Shiban et al	2015	Germany	Spider phobia	40	VR with both conditioned stimuli (CS) with aversive reinforcement in terms of air blast and scream (CS+) without aversive reinforcement (CS-)	eMagin Z800, Logitech Extreme 3D Pro Joystick
Shiban et al	2015	Germany	Spider phobia	58	VR single stimulus and single context, VR multiple stimuli and single context, VR single stimulus and multiple contexts, VR multiple stimuli and multiple scenario	eMagin Z800
Shiban et al	2015	Germany	Spider phobia	31	VRET: standard extinction, gradual extinction, Unconditioned stimuli,	eMagin Z800

					conditioned stimuli	
Urech et al	2015	Switzerland	Social Anxiety	15	Attention bias training with VR	nVisor SX60,
Webb et al	2015	USA	PTSD	58	VRE Physiological reactions measured to VR stimuli	eMagin Z800
Won et al	2016	USA	Social Anxiety	57	VRE to track head movements	nVisor sx60,

*SAD = Social anxiety disorder

*PTSD = Post traumatic stress disorder

*OCD = Obsessive compulsive disorder

<u>Depression/Stress Studies (n=4)</u>

Cikajlo	2017	Slovenia	Stress	8	Mindfulness	GearVR
Falconer et al	2016	UK	Depression	15	Virtual Bodies, adult and child	nVisor Sx111
Shah et al	2015	Singapore	Stress	22	VR DE-STRESS	ITG-PCX3 HMD,
Shiban et al	2016	Germany	Stress	45	In vivo, Tier social stress test (TSST), VRTSST, VRTSST with a virtual competitor	nVisor Sx60

<u>Pain Management (n=22)</u>

Amniabadi et al	2012	Iran	Pain and anxiety during dental treatment	120 (children)	Restorative treatment with and without vr eye glasses	i-glasses 920HR

Bahart	2015		Chronic neck pain	32	Kinematic training vs VR kinematic training	Vuzix 1200VR
Bolte et al	2014	Germany	Chronic back pain, Kinesiophobia	17	Gain-based visual feedback modification	Sensics zsight
Chen et al	2014	USA	Kinesiophobia	10	Target aiming task with altered visual feedback	Oculus Rift
Czub	2012	Poland	Analgesia	32	Heat stimulus while in a VE	E-Magin z800
Czub	2014	Poland	Analgesia	35	Memory influence during thermal pain stimulus	E-Magin z800
Czub	2014	Poland	Analgesia	30	Thermal Stimulus	E-Magin z800
Fabar	2013	The Netherlands	Pain Distraction for burns	36	VR distraction	Cybermind Hi-Res900$_{ST}$™ - 3D
Garrett	2017	Canada	Chronic pain	10	VR therapy	Oculus Rift DK2

Harvie	2015	Australia	Neck Pain	24	Visual proprioceptive cues	Oculus Rift
Hoffman	2014	USA	Pediatric burns	1	Occupational therapy with and without VR	Oculus Rift
Hua	2015	China	Lower limb Chronic wound	65	VR distraction	E-Magin z800
Jeffs	2016	USA	Burn wound care	30	Standard care, passive distraction and VR	Kaiser Optics SR80a VR helmet with SXGA
Kipping	2012	Australia	Burn Wound Care	41	VR distraction	eMagin z800
Piskorz	2014	Poland	Cold Pressor	31	VR distraction tasks at different levels of complexity	Emagin z800
Sano	2015	Japan	Phantom Limb pain	6	Reaching task in VR	Oculus Rift
Sil	2014	USA	Cold pressor pain	62	VR distraction	5DT HMD

Spyridonis	2012	UK	Pain in wheel chair users	7	Paindroid pain management	Vuzix 920
Tanja-Dijkstra	2017	UK	Cold Pressor and Dental Pain	155 (85 and 75 over two studies)	VR coastal walk	Vuzix VR920
Tashkian	2017	USA	Pain in hospitalised patients	100	3D VR vs 2D distraction video	Samsung Gear VR
Wake	2015	Japan	Phantom Limb pain	5	Visual limb distraction	Vuzix 1200 VR
Walker et al	2014	USA	Cystoscopy	45	VR distraction	5DT

<u>Addiction Studies (n=9)</u>

Acker and Mackillop	201 3	USA	Cigarette Addiction	47	VR-CET	eMagin z800
Bordnick et al	201 2	USA	Nicotine Dependence	46	NRT (Nicotine replacement Therapy), VRST (Virtual reality skills training)	eMagin Z800.
Bouchard et al	201 7	Canada	Gambling addiction	64	VRCBT and CBT	nVisor SX 60
Giovancarli et al	201 6	France	Tabacco Consumption	120	VRET, CBT	Sensics ZSight HMD
Hadley et al	201 4	USA	Adolescent Risk Reduction	42	VRE	eMagin z800
Lister et al	201	USA	Gambling	121	VR Casino	eMagin Z800

	6		Addiction		(Rigged)	
Pericot – Valverde et al	2015	Spain	Cigarette Addiction	41	VR-CET (Cue Exposure	Vuzix iWear VR920
Pericot – Valverde et al	2014	Spain	Cigarette Addiction	48	VR-CET	Vuzix iWear VR920
Thompson-Lake et al	2015	USA	Cigarette Addiction	36	VR-CET	eMagin z800

Eating Disorders/Body image (n=5)

Gutierrez – Maldonado et al	2016	Spain	Eating Disorder	113	VRCE (Virtual Reality Craving Exposure)	Oculus Rift VR HMD DK1, Computer with 3D generated images viewed with polarised glasses
Keizer et al	2016	The Netherlands	Anorexia Nervosa.	59	VR Full Body Illusion	Oculus Rift DK2
Marco et al	2013	Spain	eating disorder (bulimia nervosa, eating disorder NOS	34	VR CBT, controlled CBT	V6 by Virtual Research
Mountford et al	2015	UK	Body Image Disturbance (BID)	41	VR to elicit and detect BID	nVisor SX111,
Purvis et al	2015	USA	Body image disturbance (BID)	78	VR to elicit and detect BID	nVisor SX111 ,

ANXIETY

Anderson PL, Price M, Edwards SM, Obasaju MA, Schmertz SK, Zimand E, Calamaras MR. Virtual reality exposure therapy for social anxiety disorder: a randomized controlled trial. Journal of consulting and clinical psychology. 2013 Oct;81(5):751.

Aymerich-Franch L, Kizilcec RF, Bailenson JN. The relationship between virtual self similarity and social anxiety. Frontiers in human neuroscience. 2014;8.

Bouchard S, Dumoulin S, Robillard G, Guitard T, Klinger É, Forget H, Loranger C, Roucaut FX. Virtual reality compared with in vivo exposure in the treatment of social anxiety disorder: a three-arm randomised controlled trial. The British Journal of Psychiatry. 2017 Apr 1;210(4):276-83.

Deppermann S, Notzon S, Kroczek A, Rosenbaum D, Haeussinger FB, Diemer J, Domschke K, Fallgatter AJ, Ehlis AC, Zwanzger P. Functional co-activation within the prefrontal cortex supports the maintenance of behavioural performance in fear-relevant situations before an iTBS modulated virtual reality challenge in participants with spider phobia. Behavioural brain research. 2016 Jul 1;307:208-17.

Dibbets P, Schulte-Ostermann MA. Virtual reality, real emotions: a novel analogue for the assessment of risk factors of post-traumatic stress disorder. Frontiers in psychology. 2015;6.

Diemer J, Domschke K, Mühlberger A, Winter B, Zavorotnyy M, Notzon S, Silling K, Arolt V, Zwanzger P. Acute anxiolytic effects of quetiapine during virtual reality exposure—A double-blind placebo-controlled trial in patients with specific phobia. European Neuropsychopharmacology. 2013 Nov 30;23(11):1551-60.

Diemer J, Lohkamp N, Mühlberger A, Zwanzger P. Fear and physiological arousal during a virtual height challenge—effects in patients with acrophobia and healthy controls. Journal of anxiety disorders. 2016 Jan 31;37:30-9.

Difede J, Cukor J, Wyka K, Olden M, Hoffman H, Lee FS, Altemus M. D-cycloserine augmentation of exposure therapy for post-traumatic stress disorder: a pilot randomized clinical trial. Neuropsychopharmacology. 2014 Apr;39(5):1052.

Freeman D, Thompson C, Vorontsova N, Dunn G, Carter LA, Garety P, Kuipers E, Slater M, Antley A, Glucksman E, Ehlers A. Paranoia and post-traumatic stress disorder in the months after a physical assault: a longitudinal study examining shared and differential predictors. Psychological medicine. 2013 Dec;43(12):2673-84.

Freeman D, Antley A, Ehlers A, Dunn G, Thompson C, Vorontsova N, Garety P, Kuipers E, Glucksman E, Slater M. The use of immersive virtual reality (VR) to predict the occurrence 6 months later of paranoid thinking and posttraumatic stress symptoms assessed by self-report and interviewer methods: A study of individuals who have been physically assaulted. Psychological assessment. 2014 Sep;26(3):841.

Glotzbach-Schoon E, Tadda R, Andreatta M, Tröger C, Ewald H, Grillon C, Pauli P, Mühlberger A. Enhanced discrimination between threatening and safe contexts in high-anxious individuals. Biological psychology. 2013 Apr 30;93(1):159-66.

Hartanto D, Kampmann IL, Morina N, Emmelkamp PG, Neerincx MA, Brinkman WP. Controlling social stress in virtual reality environments. PloS one. 2014 Mar 26;9(3):e92804.

Kampmann IL, Emmelkamp PM, Hartanto D, Brinkman WP, Zijlstra BJ, Morina N. Exposure to virtual social interactions in the treatment of social anxiety disorder: a randomized controlled trial. Behaviour research and therapy. 2016 Feb 29;77:147-56.

Levy F, Leboucher P, Rautureau G, Jouvent R. E-virtual reality exposure therapy in acrophobia: A pilot study. Journal of telemedicine and telecare. 2016 Jun;22(4):215-20.

Levy F, Leboucher P, Rautureau G, Komano O, Millet B, Jouvent R. Fear of falling: efficacy of virtual reality associated with serious games in elderly people. Neuropsychiatric disease and treatment. 2016;12:877.

Malbos E, Rapee RM, Kavakli M. A controlled study of agoraphobia and the independent effect of virtual reality exposure therapy. Australian & New Zealand Journal of Psychiatry. 2013 Feb;47(2):160-8.

Meyerbroeker K, Powers MB, Van Stegeren A, Emmelkamp PM. Does yohimbine hydrochloride facilitate fear extinction in virtual reality treatment of fear of flying? A randomized placebo-controlled trial. Psychotherapy and psychosomatics. 2012;81(1):29-37.

Morina N, Brinkman WP, Hartanto D, Emmelkamp PM. Sense of presence and anxiety during virtual social interactions between a human and virtual humans. PeerJ. 2014 Apr 3;2:e337.

Morina N, Brinkman WP, Hartanto D, Kampmann IL, Emmelkamp PM. Social interactions in virtual reality exposure therapy: A proof-of-concept pilot study. Technology and Health Care. 2015 Jan 1;23(5):581-9.

Mosig C, Merz CJ, Mohr C, Adolph D, Wolf OT, Schneider S, Margraf J, Zlomuzica A. Enhanced discriminative fear learning of phobia-irrelevant stimuli in spider-fearful individuals. Frontiers in behavioral neuroscience. 2014;8.

Mühlberger A, Neumann R, Lozo L, Müller M, Hettinger M. Bottom-up and top-down influences of beliefs on emotional responses: fear of heights in a virtual environment.

Norrholm SD, Jovanovic T, Gerardi M, Breazeale KG, Price M, Davis M, Duncan E, Ressler KJ, Bradley B, Rizzo A, Tuerk PW. Baseline psychophysiological and cortisol reactivity as a predictor of PTSD treatment outcome in virtual reality exposure therapy. Behaviour research and therapy. 2016 Jul 31;82:28-37.

Notzon S, Deppermann S, Fallgatter A, Diemer J, Kroczek A, Domschke K, Zwanzger P, Ehlis AC. Psychophysiological effects of an iTBS modulated virtual reality challenge including participants with spider phobia. Biological psychology. 2015 Dec 31;112:66-76.

Pallavicini F, Cipresso P, Raspelli S, Grassi A, Serino S, Vigna C, Triberti S, Villamira M, Gaggioli A, Riva G. Is virtual reality always an effective stressors for exposure treatments? Some insights from a controlled trial. BMC psychiatry. 2013 Feb 11;13(1):52.

Peperkorn HM, Alpers GW, Mühlberger A. Triggers of fear: perceptual cues versus conceptual information in spider phobia. Journal of clinical psychology. 2014 Jul 1;70(7):704-14.

Peperkorn HM, Diemer JE, Alpers GW, Mühlberger A. Representation of patients' hand modulates fear reactions of patients with spider phobia in virtual reality. Frontiers in psychology. 2016;7.

Powers MB, Briceno NF, Gresham R, Jouriles EN, Emmelkamp PM, Smits JA. Do conversations with virtual avatars increase feelings of social anxiety?. Journal of anxiety disorders. 2013 May 31;27(4):398-403.

Price M, Maples JL, Jovanovic T, Norrholm SD, Heekin M, Rothbaum BO. An investigation of outcome expectancies as a predictor of treatment response for combat veterans with PTSD: Comparison of clinician, self-report, and biological measures. Depression and anxiety. 2015 Jun;32(6):392.

Reger GM, Koenen-Woods P, Zetocha K, Smolenski DJ, Holloway KM, Rothbaum BO, Difede J, Rizzo AA, Edwards-Stewart A, Skopp NA, Mishkind M. Randomized controlled trial of prolonged exposure using imaginal exposure vs. virtual reality exposure in active duty soldiers with deployment-related posttraumatic stress disorder (PTSD).

Rinck M, Koene M, Telli S, Moerman-van den Brink W, Verhoeven B, Becker ES. The time course of location-avoidance learning in fear of spiders. Cognition and Emotion. 2016 Apr 2;30(3):430-43.

Rothbaum BO, Price M, Jovanovic T, Norrholm SD, Gerardi M, Dunlop B, Davis M, Bradley B, Duncan EJ, Rizzo A, Ressler KJ. A randomized, double-blind evaluation of D-cycloserine or alprazolam combined with virtual reality exposure therapy for posttraumatic stress disorder in Iraq and Afghanistan War veterans. American Journal of Psychiatry. 2014 Jun;171(6):640-8.

Rus-Calafell M, Gutiérrez-Maldonado J, Botella C, Baños RM. Virtual reality exposure and imaginal exposure in the treatment of fear of flying: A pilot study. Behavior modification. 2013 Jul;37(4):568-90.

Safir MP, Wallach HS, Bar-Zvi M. Virtual reality cognitive-behavior therapy for public speaking anxiety: one-year follow-up. Behavior modification. 2012 Mar;36(2):235-46.

Shiban Y, Peperkorn H, Alpers GW, Pauli P, Mühlberger A. Influence of perceptual cues and conceptual information on the activation and reduction of claustrophobic fear. Journal of behavior therapy and experimental psychiatry. 2016 Jun 30;51:19-26.

Shiban Y, Fruth MB, Pauli P, Kinateder M, Reichenberger J, Mühlberger A. Treatment effect on biases in size estimation in spider phobia. Biological psychology. 2016 Dec 31;121:146-52.

Shiban Y, Brütting J, Pauli P, Mühlberger A. Fear reactivation prior to exposure therapy: does it facilitate the effects of VR exposure in a randomized clinical sample?. Journal of behavior therapy and experimental psychiatry. 2015 Mar 31;46:133-40

Shiban Y, Reichenberger J, Neumann ID, Mühlberger A. Social conditioning and extinction paradigm: a translational study in virtual reality. Frontiers in psychology. 2015;6.

Shiban Y, Schelhorn I, Pauli P, Mühlberger A. Effect of combined multiple contexts and multiple stimuli exposure in spider phobia: a randomized clinical trial in virtual reality. Behaviour research and therapy. 2015 Aug 31;71:45-53.

Shiban Y, Wittmann J, Weißinger M, Mühlberger A. Gradual extinction reduces reinstatement. Frontiers in behavioral neuroscience. 2015;9.

Urech A, Krieger T, Chesham A, Mast FW, Berger T. virtual reality-based attention bias modification training for social anxiety: a feasibility and proof of concept study. Frontiers in psychiatry. 2015;6.

Webb AK, Vincent AL, Jin AB, Pollack MH. Physiological reactivity to nonideographic virtual reality stimuli in veterans with and without PTSD. Brain and behavior. 2015 Feb 1;5(2).

Won AS, Perone B, Friend M, Bailenson JN. Identifying anxiety through tracked head movements in a virtual classroom. Cyberpsychology, Behavior, and Social Networking. 2016 Jun 1;19(6):380-7.

Pain Management

Aminabadi NA, Erfanparast L, Sohrabi A, Oskouei SG, Naghili A. The impact of virtual reality distraction on pain and anxiety during dental treatment in 4-6 year-old children: a randomized controlled clinical trial. Journal of dental research, dental clinics, dental prospects. 2012;6(4):117.

Bahat HS, Takasaki H, Chen X, Bet-Or Y, Treleaven J. Cervical kinematic training with and without interactive VR training for chronic neck pain–a randomized clinical trial. Manual therapy. 2015 Feb 1;20(1):68-78.

Bolte, B. Virtual reality system for the enhancement of mobility in patients with chronic back pain. International Journal of Child Health and Human Development. 2016 Jul 1;9(3):305.

Chen KB, Ponto K, Sesto ME, Radwin RG. Influence of altered visual feedback on neck movement for a virtual reality rehabilitative system. InProceedings of the Human Factors and Ergonomics Society Annual Meeting 2014 Sep (Vol. 58, No. 1, pp. 693-697). Sage CA: Los Angeles, CA: SAGE Publications.

Czub M, Piskorz J. Effectiveness of Different Virtual Reality Environ-ments on Thermal Pain Distraction. Polish Journal of Applied Psychology. 2012;10(2):7-19.

Czub M, Piskorz J, Misiewicz M, Hodowaniec P, Mrula M, Urbańska K. Influence of memory on experienced pain during Virtual Reality analgesia. Polish Journal of Applied Psychology. 2014 Dec 1;12(4):41-52

Czub M, Piskorz J. How body movement influences virtual reality analgesia?. InInteractive Technologies and Games (iTAG), 2014 International Conference on 2014 Oct 16 (pp. 13-19). IEEE.

Faber AW, Patterson DR, Bremer M. Repeated use of immersive virtual reality therapy to control pain during wound dressing changes in pediatric and adult burn patients. Journal of Burn Care & Research. 2013 Sep 1;34(5):563-8.

Garrett B, Taverner T, McDade P. Virtual Reality as an Adjunct Home Therapy in Chronic Pain Management: An Exploratory Study. JMIR medical informatics. 2017 Apr;5(2).

Gromala D, Tong X, Choo A, Karamnejad M, Shaw CD. The virtual meditative walk: virtual reality therapy for chronic pain management. InProceedings of the 33rd Annual ACM Conference on Human Factors in Computing Systems 2015 Apr 18 (pp. 521-524). ACM.

Harvie DS, Broecker M, Smith RT, Meulders A, Madden VJ, Moseley GL. Bogus visual feedback alters onset of movement-evoked pain in people with neck pain. Psychological Science. 2015 Apr;26(4):385-92.

Hoffman HG, Meyer III WJ, Ramirez M, Roberts L, Seibel EJ, Atzori B, Sharar SR, Patterson DR. Feasibility of articulated arm mounted Oculus Rift Virtual Reality goggles for adjunctive pain control during occupational therapy in pediatric burn patients. Cyberpsychology, Behavior, and Social Networking. 2014 Jun 1;17(6):397-401.

Hua Y, Qiu R, Yao WY, Zhang Q, Chen XL. The effect of virtual reality distraction on pain relief during dressing changes in children with chronic wounds on lower limbs. Pain Management Nursing. 2015 Oct 1;16(5):685-91.

Jeffs D, Dorman D, Brown S, Files A, Graves T, Kirk E, Meredith-Neve S, Sanders J, White B, Swearingen CJ. Effect of virtual reality on adolescent pain during burn wound care. Journal of Burn Care & Research. 2014 Sep 1;35(5):395-408.

Kipping B, Rodger S, Miller K, Kimble RM. Virtual reality for acute pain reduction in adolescents undergoing burn wound care: a prospective randomized controlled trial. Burns. 2012 Aug 1;38(5):650-7.

Piskorz J, Czub M. Distraction of attention with the use of virtual reality. Influence of the level of game complexity on the level of experienced pain. Polish Psychological Bulletin. 2014 Dec 1;45(4):480-7.

Sano Y, Ichinose A, Wake N, Osumi M, Sumitani M, Kumagaya SI, Kuniyoshi Y. Reliability of phantom pain relief in neurorehabilitation using a multimodal virtual reality system. InEngineering in Medicine and Biology Society (EMBC), 2015 37th Annual International Conference of the IEEE 2015 Aug 25 (pp. 2482-2485). IEEE.

Sil S, Dahlquist LM, Thompson C, Hahn A, Herbert L, Wohlheiter K, Horn S. The effects of coping style on virtual reality enhanced videogame distraction in children undergoing cold pressor pain. Journal of behavioral medicine. 2014 Feb 1;37(1):156-65.

Spyridonis F, Grønli TM, Hansen J, Ghinea G. Evaluating the usability of a virtual reality-based Android application in managing the pain experience of wheelchair users. InEngineering in Medicine and Biology Society (EMBC), 2012 Annual International Conference of the IEEE 2012 Aug 28 (pp. 2460-2463). IEEE.

Tanja-Dijkstra K, Pahl S, White MP, Auvray M, Stone RJ, Andrade J, May J, Mills I, Moles DR. The Soothing Sea: A Virtual Coastal Walk Can Reduce Experienced and Recollected Pain. Environment and Behavior. 2017:0013916517710077.

Tashjian VC, Mosadeghi S, Howard AR, Lopez M, Dupuy T, Reid M, Martinez B, Ahmed S, Dailey F, Robbins K, Rosen B. Virtual reality for management of pain in hospitalized patients: results of a controlled trial. JMIR mental health. 2017 Jan;4(1).

Wake N, Sano Y, Oya R, Sumitani M, Kumagaya SI, Kuniyoshi Y. Multimodal virtual reality platform for the rehabilitation of phantom limb pain. InNeural Engineering (NER), 2015 7th International IEEE/EMBS Conference on 2015 Apr 22 (pp. 787-790). IEEE.

Walker MR, Kallingal GJ, Musser JE, Folen R, Stetz MC, Clark JY. Treatment efficacy of virtual reality distraction in the reduction of pain and anxiety during cystoscopy. Military medicine. 2014 Aug 1;179(8):891-6.

DEPRESSION/STRESS

Cikajlo, Imre, Ursa Cizman Staba, Suzana Vrhovac, Frances Larkin, and Mark Roddy. "A Cloud-Based Virtual Reality App for a Novel Telemindfulness Service: Rationale, Design and Feasibility Evaluation." *JMIR research protocols* 6, no. 6 (2017).

Falconer CJ, Rovira A, King JA, Gilbert P, Antley A, Fearon P, Ralph N, Slater M, Brewin CR. Embodying self-compassion within virtual reality and its effects on patients with depression. British Journal of Psychiatry Open. 2016 Jan 1;2(1):74-80.

Shah LB, Torres S, Kannusamy P, Chng CM, He HG, Klainin-Yobas P. Efficacy of the virtual reality-based stress management program on stress-related variables in people with mood disorders: the feasibility study. Archives of psychiatric nursing. 2015 Feb 28;29(1):6-13.

Shiban Y, Diemer J, Brandl S, Zack R, Mühlberger A, Wüst S. Trier social stress test in vivo and in virtual reality: Dissociation of response domains. International Journal of Psychophysiology. 2016 Dec 31;110:47-55.

ADDICTION

Acker J, MacKillop J. Behavioral economic analysis of cue-elicited craving for tobacco: a virtual reality study. nicotine & tobacco research. 2013 Jan 15;15(8):1409-16..

Bordnick PS, Traylor AC, Carter BL, Graap KM. A feasibility study of virtual reality-based coping skills training for nicotine dependence. Research on social work practice. 2012 May;22(3):293-300.

Bouchard S, Robillard G, Giroux I, Jacques C, Loranger C, St-Pierre M, Chrétien M, Goulet A. Using Virtual reality in the Treatment of gambling Disorder: The Development of a new Tool for cognitive Behavior Therapy. Frontiers in psychiatry. 2017;8.

Giovancarli C, Malbos E, Baumstarck K, Parola N, Pélissier MF, Lançon C, Auquier P, Boyer L. Virtual reality cue exposure for the relapse prevention of tobacco consumption: a study protocol for a randomized controlled trial. Trials. 2016 Feb 19;17(1):96

Hadley W, Houck CD, Barker DH, Garcia AM, Spitalnick JS, Curtis V, Roye S, Brown LK. Eliciting Affect via Immersive Virtual Reality: A Tool for Adolescent Risk Reduction. Journal of pediatric psychology. 2013 Dec 23;39(3):358-68.

Lister JJ, Nower L, Wohl MJ. Gambling goals predict chasing behavior during slot machine play. Addictive behaviors. 2016 Nov 30;62:129-34.

Pericot-Valverde I, García-Rodríguez O, Gutiérrez-Maldonado J, Secades-Villa R. Individual variables related to craving reduction in cue exposure treatment. Addictive behaviors. 2015 Oct 31;49:59-63.

Pericot-Valverde I, Secades-Villa R, Gutiérrez-Maldonado J, García-Rodríguez O. Effects of systematic cue exposure through virtual reality on cigarette craving. nicotine & tobacco research. 2014 Jun 23;16(11):1470-7.

Thompson-Lake DG, Cooper KN, Mahoney III JJ, Bordnick PS, Salas R, Kosten TR, Dani JA, De La Garza R. Withdrawal symptoms and nicotine dependence severity predict virtual reality craving in cigarette-deprived smokers. Nicotine & Tobacco Research. 2014 Dec 4;17(7):796-802..

EATING DISORDERS

Gutiérrez-Maldonado J, Pla-Sanjuanelo J, Ferrer-García M. Cue-exposure software for the treatment of bulimia nervosa and binge eating disorder. Psicothema. 2016;28(4).

Keizer A, van Elburg A, Helms R, Dijkerman HC. A virtual reality full body illusion improves body image disturbance in anorexia nervosa. PloS one. 2016 Oct 6;11(10):e0163921.

Marco JH, Perpina C, Botella C. Effectiveness of cognitive behavioral therapy supported by virtual reality in the treatment of body image in eating disorders: one year follow-up. Psychiatry Research. 2013 Oct 30;209(3):619-25.

Mountford VA, Tchanturia K, Valmaggia L. “What Are You Thinking When You Look at Me?” A Pilot Study of the Use of Virtual Reality in Body Image. Cyberpsychology, Behavior, and Social Networking. 2016 Feb 1;19(2):93-9.

Purvis CK, Jones M, Bailey JO, Bailenson J, Taylor CB. Developing a novel measure of body satisfaction using virtual reality. PloS one. 2015 Oct 15;10(10):e0140158.
