# Supplementary material for: Head-Mounted Virtual Reality and Mental Health: Critical Review of Current Research
Source: JMIR Serious Games. 2018 Jul 6;6(3):e14. doi: 10.2196/games.9226 (PMC6054705; doi:10.2196/games.9226)
Supplement: Multimedia Appendix 2 [file games_v6i3e14_app2.pdf]

Type	Resolution	Hz	Field of View (diagonal unless stated otherwise)	Number of Studies
eMagin z800	800 x 600	60 Hz	40°D	34
nVisor SX60	1280 x 1024	60Hz	60°D	8
nVisor SX111	1280 x 1024	60Hz	111°D	4
nVisor ST50	1280 x 1024	60 Hz	50°D	1
Sony HMZ-T1	1280 x 720	60 Hz	51.6°D	1
5DT HMD	800 x 600	-	40°D	3
VisuaStim	1280 x 1024	85 Hz	40°D	1
Kaiser XL 50	1024 x 768	60 Hz	50°D	1
VR1280	1280 x 1024	60 Hz	60°D	1
Virtual Realities VR HMD pro 3D-42	800 x 600	-	42 (assumed diagonal)	1
Cybermind Visette Pro	640 x 480	60 Hz	71.5°D	2
Vuzix iWear VR920	640 x 480	-	-	5
Vuzix VR1200	-	-	-	2
VFX3D	640 x 480	-	35°D	1
Sensis Zsight	1280 x 1024	60Hz	60°D	2
V6 by Virtual Research Systems	640 x 480	60Hz	60°D	1
V8 by Virtual Research Systems	640x480	60Hz	60	1
Oculus Rift DK1	640 x 800	60Hz	110°D	1
Oculus Rift DK2	960 x 1080	75Hz	100°D	6
ITV goggles ITG Wideview Xl edition	-	-	-	1
Samsung Gear VR	2560 x 1440 * (Super AMOLED and dependent on smartphone used)	60 Hz	96 °D	1
i-glasses 920HR	-	-	35°D	1
Kaiser Optics SR80a	-	-	-	1

TABLE 2 – Head Mounted Display Specifications

* AMOLED (active-matrix organic light-emitting diode)

Below: A comparison of capable HMDs commonly sold on the market today.

HTC Vive	2160 x 1200	90 Hz	110°D	-
HTC Vive Pro	2880 x 1600	90Hz	110°D	-
Playstaion VR	1920 x 1080	90Hz (120Hz in cinema mode)	100°D (approximately)	-
Samsung Odyssey	1,440 x 1,600 per screen	90 – 60 Hz	110°D	-
